# Relationships of Linear and Non-linear Measurements of Post-stroke Walking Activity and Their Relationship to Weather

**DOI:** 10.3389/fspor.2020.551542

**Published:** 2020-11-03

**Authors:** Sydney C. Andreasen, Tamara R. Wright, Jeremy R. Crenshaw, Darcy S. Reisman, Brian A. Knarr

**Affiliations:** ^1^Department of Biomechanics, Biomechanics Research Building, University of Nebraska at Omaha, Omaha, NE, United States; ^2^Clinical Research Laboratory, Department of Physical Therapy, University of Delaware, Newark, DE, United States; ^3^Falls and Mobility Laboratory, Department of Kinesiology and Applied Physiology, University of Delaware, Newark, DE, United States; ^4^Neuromotor Behavior Lab, Department of Physical Therapy, University of Delaware, Newark, DE, United States

**Keywords:** stroke, physical activity, structure, complexity, weather, precipitation

## Abstract

**Background:** Stroke survivors are more sedentary than the general public. Previous research on stroke activity focuses on linear quantities. Non-linear measures, such as Jensen-Shannon Divergence and Lempel-Ziv Complexity, may help explain when and how stroke survivors move so that interventions to increase activity may be designed more effectively.

**Objectives:** Our objective was to understand what factors affect a stroke survivor's physical activity, including weather, by characterizing activity by step counts, structure, and complexity.

**Methods:** A custom MATLAB code was used to analyze clinical trial (NCT02835313, https://clinicaltrials.gov/ct2/show/NCT02835313) data presented as minute by minute step counts. Six days of data were analyzed for 142 participants to determine the regularity of activity structure across days and complexity patterns of varied cadences. The effect of steps on structure and complexity, the season's effect on steps, structure, and complexity, and the presence of precipitation's effect on steps and complexity were all analyzed.

**Results:** Step counts and regularity were linearly related (*p* < 0.001). Steps and complexity were quadratically related (*r*^2^ = 0.70 for mean values, 0.64 for daily values). Season affected complexity between spring and winter (*p* = 0. 019). Season had no effect on steps or structure. Precipitation had no effect on steps or complexity.

**Conclusions:** Stroke survivors with high step counts are active at similar times each day and have higher activity complexities as measured through patterns of movement at different intensity levels. Non-linear measures, such as Jensen-Shannon Divergence and Lempel-Ziv Complexity, are valuable in describing a person's activity. Weather affects our activity parameters in terms of complexity between spring and winter.

## Introduction

People who have survived stroke are known to be more sedentary than otherwise healthy individuals (Sjöholm et al., 2014), and inactivity increases health risks including mortality (Proper et al., 2011; Thorp et al., 2011), cardiovascular disease (Warburton et al., 2006; De Rezende et al., 2014), diabetes (Warburton et al., 2006), cancer (Warburton et al., 2006), osteoporosis and arthritis (Warburton et al., 2006), depression (Warburton et al., 2006), and recurrent stroke (Mohan et al., 2011). Importantly, one can increase their physical activity levels and decrease their risk of the previously mentioned diseases (Warburton et al., 2006). Therefore, it is pertinent that we understand physical activity and the factors that affect physical activity in stroke survivors, in order to best promote its increase.

To our knowledge, only a few rehabilitation intervention studies have examined changes in physical activity as an outcome of the intervention (Pang et al., 2005; Michael et al., 2009; Mirelman et al., 2009; Mudge et al., 2009; Moore et al., 2010). When activity is objectively measured, as opposed to self-reported data that may show positively skewed activity levels (Resnick et al., 2008), the results indicated that current rehabilitation interventions have limited impact on the daily walking activity of chronic stroke survivors (Pang et al., 2005; Michael et al., 2009; Mirelman et al., 2009; Mudge et al., 2009; Moore et al., 2010). These studies found either no improvement (Pang et al., 2005; Michael et al., 2009; Mudge et al., 2009), or when an improvement was observed, the participants still remained relatively sedentary after the intervention, walking fewer steps per day than sedentary older adults and well below the recommended levels of walking activity (Pang et al., 2005; Moore et al., 2010). Recent work has shown that giving weekly physical activity goals to stroke survivors can help to improve their overall physical activity levels (Danks et al., 2014). Furthermore, the use of a simple interactive mobile application has been found to be a strong facilitator of the increase of physical activity through step goals (Paul et al., 2016). These approaches primarily target the *quantity* of physical activity. Other popular platforms, however, more comprehensively target the type, timing, intensity, and regularity of physical activity (Conroy et al., 2014). This approach not only addresses the *quantity* of physical activity, but its day-to-day *structure* (i.e., the distribution of activity throughout the day) and *complexity* (i.e., the variations in length and intensity of activity). The interplay between the quantity and structure and complexity of physical activity, however, is not well-established in those with chronic stroke. By quantifying physical activity structure and complexity and their relationship to physical activity quantity, we can better understand the barriers to physical activity, determine the underlying mechanisms of how physical activity quantity can be improved and sustained, and identify new intervention targets for increasing physical activity in stroke survivors.

Physical activity structure and complexity characterize an individual's movement, including when a person moves, how regularly they move, and how intense their movements are. Though the topic of physical activity structure is not unexplored, many studies rely on linear measures to quantify physical activity, such as steps per day (Eng and Tang, 2007; Sumukadas et al., 2009; Roos et al., 2012; Knarr et al., 2013; Danks et al., 2014), total time walking (Roos et al., 2012; Knarr et al., 2013; Danks et al., 2014), and bouts of physical activity per day (Roos et al., 2012; Knarr et al., 2013; Danks et al., 2014). Scientific literature is lacking analyses of physical activity after stroke using non-linear measures. While linear parameters can provide valuable physical activity information, it is important to consider non-linear measures to more completely capture the complex aspects of physical activity. Such non-linear measures provide insight into how a person's physical activity is organized into their daily life (Paraschiv-Ionescu et al., 2008). For example, Jensen-Shannon Divergence (JSD) is an entropy-based non-linear measure that can be used to analyze a person's activity over multiple days. JSD describes data with a measure of divergence from the data's probability distribution (Lin, 1991). JSD has been previously used in studies of behavior relating to the patterns in which individuals make phone calls (Saramaki et al., 2014; Aledavood et al., 2015). We can apply JSD to physical activity by comparing the variability of each minute's step counts to their average activity at that time of day over multiple days. A second non-linear analysis named Lempel-Ziv Complexity (LZC) analyzes sequences of data and their occurrences as patterns. This measure provides a description of data through the number of distinct patterns as well as how often each occurs (Aboy et al., 2006). Previous studies have used LZC to analyze the complexities of signals like those in EEG or ECG studies (Abásolo et al., 2006; Aboy et al., 2006). One study used a combination of type, duration, and intensity of physical activity to characterize their complexity measure, which showed that “well-functioning older adults” who had greater psychological concerns about falling had lower complexity in their activity (Paraschiv-Ionescu et al., 2018). The same concept can be applied to the patterns that occur in step counts after stroke. For example, patterns may occur as a person moves in different levels of intensity of activity, as measured by cadence. LZC can then analyze how these patterns occur throughout the day; more distinct patterns as well as a greater number of occurrences of those patterns, contribute to a higher level of complexity.

A combination of linear and non-linear analyses would allow for investigating overall physical activity. Linearly, this could include the number of steps taken in a day and the number of discrete instances that a person is active. Non-linear measures may be the structure of a person's physical activity through their patterns of activity and the variances in types of activities that occur in those patterns. These analyses could provide a more detailed description of how and when people move in order to show what aspects of their physical activity structure that could be targeted for intervention.

To properly design an intervention to promote physical activity, it is not only important to describe activity amount, structure, and complexity; it is necessary to determine any barriers that may prevent an individual from being physically active. One factor that has been quantitatively reported to affect healthy adult physical activity (Jones et al., 2017) and qualitatively reported to affect stroke physical activity is weather, which is perceived as a barrier to physical activity quantity when it is unfavorable. This psychological barrier is especially present with precipitation, as it introduces a perceived risk of falling (Törnbom et al., 2017). While qualitative reports of barriers are valuable, quantitative studies can determine whether or not any perceived barrier translates into a clinically significant barrier to activity that an individual actually performs. Quantitative studies have not reached a consensus on the relationship between weather parameters and physical activity, and there is a lack of quantitative investigations on the effect of weather in those with stroke in particular (Tucker and Gilliland, 2007). It is important to understand the relationship between weather and physical activity after stroke to determine whether this is a substantial barrier that needs to be addressed in interventions aimed at improving physical activity after stroke, such as through substituted indoor exercises when poor weather may introduce a barrier to feasibly achieving adequate activity amounts.

The purpose of this study is to examine physical activity patterns after stroke and the relationship of these patterns to weather parameters. The goal is to use the information generated from this study to inform further development of physical activity promotion programs that result in the greatest increase in physical activity after stroke. We will explore the relationship between the number of steps a stroke survivor takes in a day and the structure and complexity of their physical activity as measured by JSD and LZC, respectively. If a significant relationship is found, then these non-linear measures may be used as a predictor of activity in stroke survivors. We hypothesize that, in persons post-stroke, physical activity amounts will increase and become more similarly structured and complex across days during seasons with more favorable weather, such as spring, where temperatures are mild to warm and any precipitation is less likely to produce an icy walking surface than in winter, as well as over days with no precipitation. In contrast, physical activity will decrease and become less regularly patterned and less complex during unfavorable weather conditions, like cold icy seasons, as well as across days with precipitation.

## Methods

**Figure 1:**
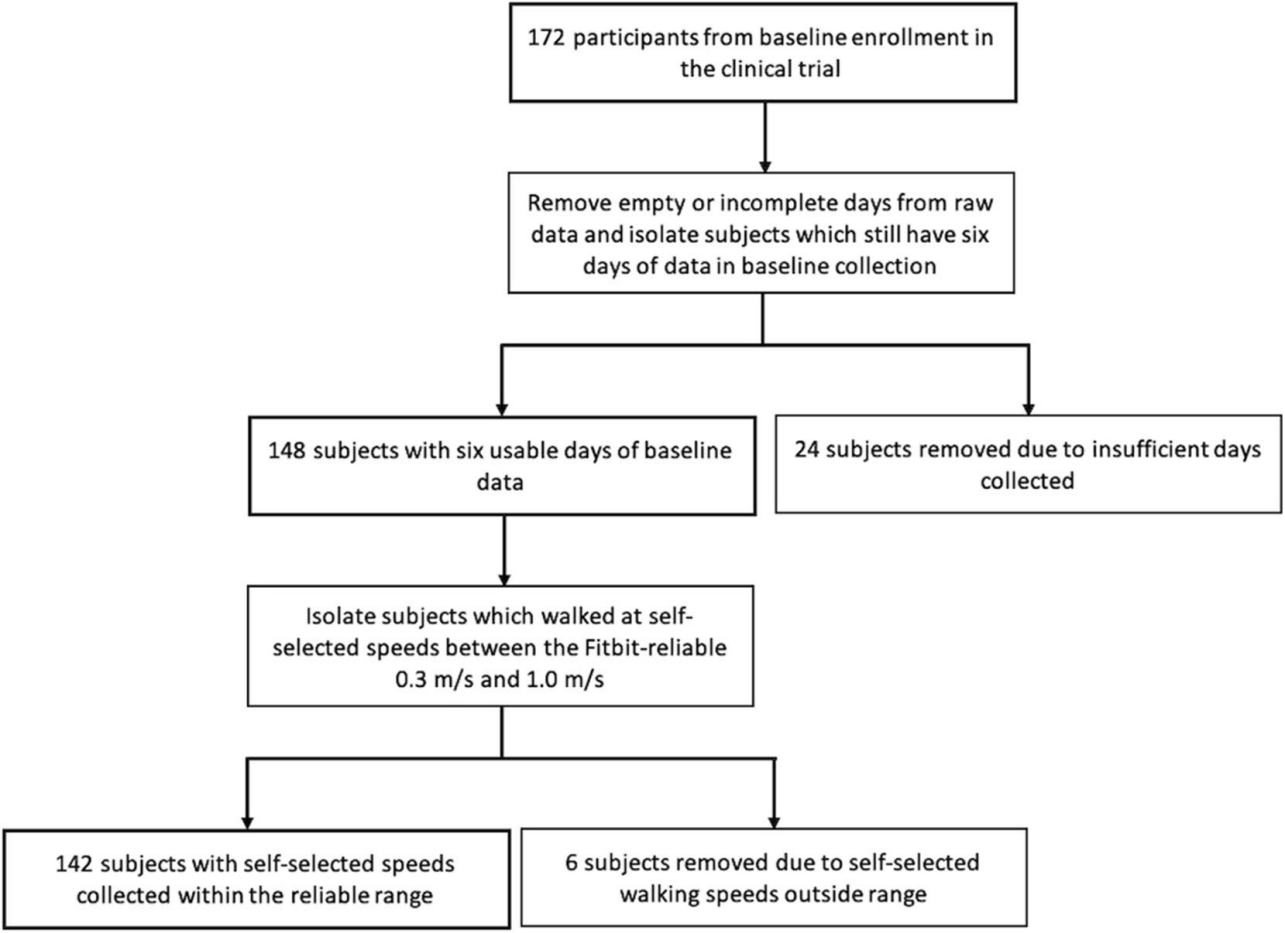
Illustrating the decision process for including subjects in analysis.

Physical activity data was collected as part of a larger clinical trial (NCT02835313) performed through the University of Delaware. All subjects provided written informed consent, with the study protocol approved by the University of Delaware Human Subjects Review Board. Participants with chronic (including single or multiple) stroke(s) to either hemisphere(s), age 21–85, with the ability to walk at a self-selected walking speed >0.3 m/s and <1.0 m/s were included. Ankle-foot orthoses, canes, and walkers were allowed, but participants must be capable of walking without additional human assistance (Wright et al., 2018). Exclusion criteria included evidence of cerebellar stroke, other potentially disabling neurological conditions, lower limb Botox injection within fourth months, current participation in physical therapy, inability to walk outside the home before stroke, coronary artery bypass graft or stent replacement or heart attack within 3 months, or musculoskeletal pain which limits activity (Wright et al., 2018). Physical activity is defined as “bodily movement produced by skeletal muscles that results in energy expenditure” (2018 Physical Activity Guidelines Advisory Committee, 2018). Participants wore a Fitbit (Fitbit One or Zip) on their less-affected ankle, which recorded their routine activity step counts at 1 min intervals during all waking hours, except swimming and bathing, without feedback on activity from the device. Fitbit monitors have been found to be reliable and accurate for monitoring activity data above 0.3 m/s (Fulk et al., 2014; Klassen et al., 2016, 2017; Hui et al., 2018). Data was collected for 172 participants after their initial study visit, with those outside the reliable walking speed range being excluded afterward; the present data was collected as pre-intervention baseline data for all participants. The goal in the clinical trial was for subjects to have 7 days of useable data (Wright et al., 2018), so the final number of days in the initial collection period varied depending on the participant. A custom MATLAB code was utilized to analyze the activity data (MathWorks, Natick, MA, USA). See Figure 1 illustrating which subjects' data were analyzed.

First, we removed partially recorded days and days that recorded no physical activity from our data set. Though all trial participants were contacted daily during activity monitoring as a reminder to wear the Fitbit (Wright et al., 2018), not every participant wore the device every day. In total, 142 of the participants had step data collected for at least 6 entire days at reliable speeds. Six days was determined adequate for characterizing a person's activity patterns (Hart et al., 2011; Dillon et al., 2016), and therefore, the first 6 useable days were selected for further analysis. These 6 days were either continuous or within the same 7 day period for each participant; if an individual's analyzed data spanned seven calendar days, this was due to a single day of zero data being excluded from the middle.

Total daily steps for each day and each participant (*n* = 852) were determined by summing all step counts recorded in the respective day. The mean daily steps of each participant (*n* = 142) was determined by taking the average of their total daily steps over 6 days.

In addition to traditional measures, two non-linear measures were used to describe the patterns embedded in participant's physical activity and determine the relationship between non-linear structure and complexity and number of steps. Jensen-Shannon Divergence (JSD) is a non-linear analysis based on inequality and entropy (Lin, 1991). JSD was used to determine the similarity, across 6 days, of each participant's minute-to-minute physical activity patterns by developing a probability distribution of activity throughout one's day and determining the divergence of data from the distribution. Low JSD values correspond to low physical activity entropy. In other words, stroke survivors with more similar physical activity patterns across days will have lesser JSD values. Lempel-Ziv Complexity (LZC), which analyzed the number of distinct patterns, which were created by participants transitioning from one level of activity intensity to another, and how often each pattern recurred (Aboy et al., 2006), determined daily physical activity complexity values. Low, low-medium, medium, and high intensity physical activity were defined as ranges of cadences as <15 steps per minute, ≥15 and <30 steps per minute, ≥30 and <45 steps per minute, and ≥45 steps per minute, respectively (Manns and Baldwin, 2009). Greater combinations of these cadence ranges as well as greater occurrences of each combination will contribute to higher daily complexity values (Paraschiv-Ionescu et al., 2012).

Recorded weather data was retrieved from the Philadelphia Mt. Holly Weather Forecast Office in New Jersey via the National Centers for Environmental Information (NCEI) and was used for all subjects, given that subjects were recruited from the northern Delaware and Philadelphia metropolitan area. Seasons were defined by 3 month sequences based on winter as the December through February months. To analyze the relationship between activity and weather, subjects' mean daily steps across 6 days, JSD values (*n* = 142), and daily LZC values (*n* = 852) were each categorized into seasons. Further total daily steps and LZC values for each individual day (*n* = 852) were categorized by having, or not having, precipitation in each respective day to determine the effect of precipitation as a barrier on physical activity.

**Figure 2:**
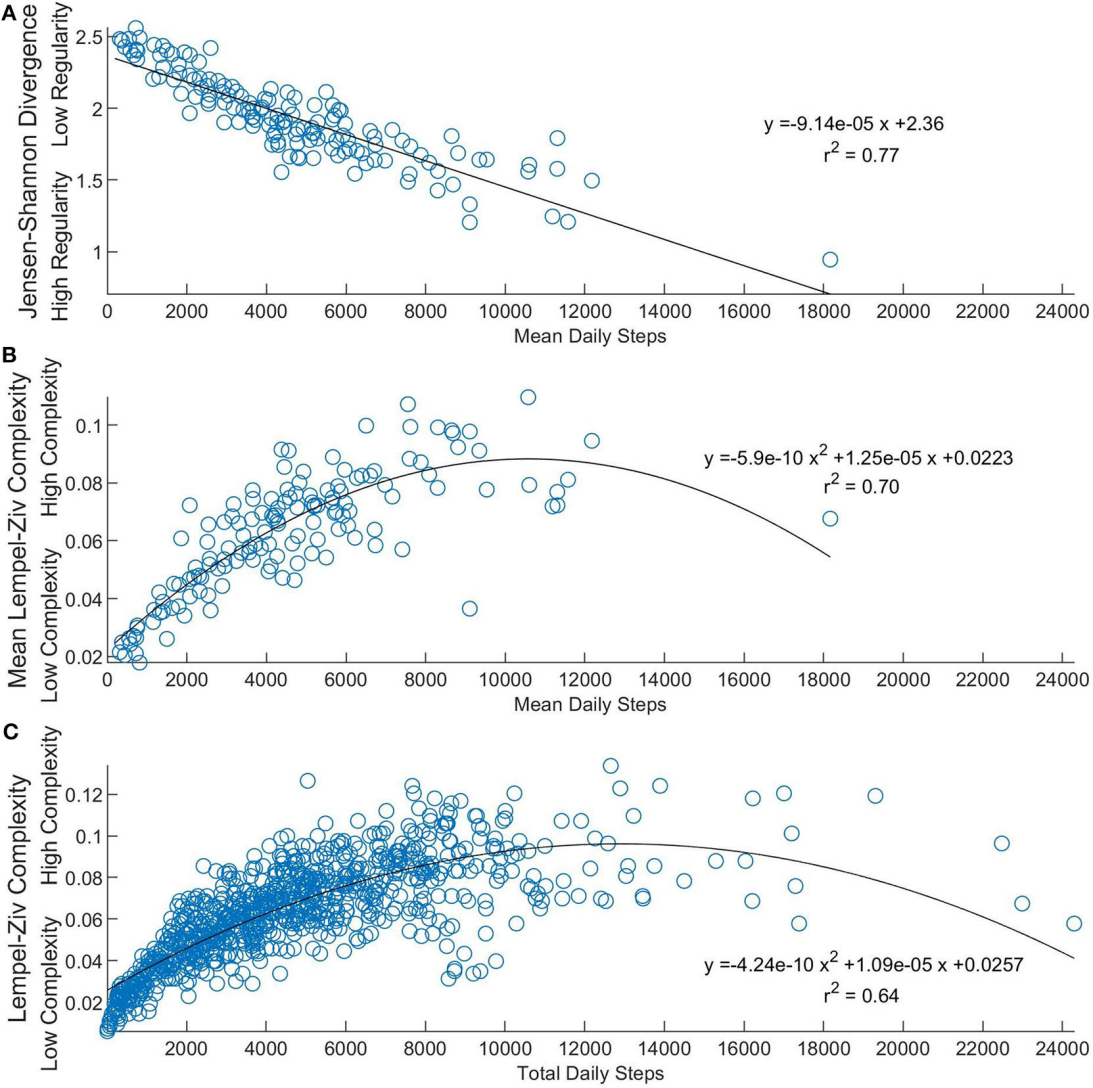
Relating linear and nonlinear physical activity measures. **(A)** Shows the relationship between JSD values and mean daily steps over 6 days (*n* = 142). Lesser JSD values represent more similarity in physical activity patterns across days. **(B)** Compares participants' average activity complexity value to their mean daily steps value (*n* = 142). **(C)** Shows the relationship between daily LZC values and total daily steps (*n* = 852). Lesser LZC values represent less complex physical activity patterns.

Statistical analysis was performed using SPSS 26.0 (IBM, Armonk, NY, USA). First, a possible relationship between the number of steps a stroke survivor takes in a day and the structure and complexity of their activity was explored. Linear regressions were used to quantify the relationship between mean daily steps to JSD values. A quadratic regression determined the relationship between total daily steps and LZC values, as well as mean daily steps to mean LZC values. A quadratic relationship was used based on the following observations and reasoning: (1) a non-linear relationship was evident from scatterplots of total steps and complexity (Figure 2), (2) it is logical that the potential influence of complexity on step counts is robust at low-to-moderate physical activities, with a diminishing effect for those with substantially high physical activity levels, and (3) theoretically, at a point of extremely high physical activity, there is little freedom to have complex activity behavior. Then, analysis of variance tests were used to test our hypothesis that (1) activity amount, structure, and complexity would increase in favorable seasons and periods of no precipitation while (2) activity amount, structure, and complexity would decrease in seasons, which on average, have less favorable weather, and periods with precipitation. One-way ANOVA tests were used to analyze the effect of season on mean daily steps, JSD, and LZC values. One-way ANOVA tests were also used to analyze the effect of rain compared to no rain on total daily steps and LZC values. Relationships with *p* < 0.05 and *r*^2^ > 0.49 were considered significant and meaningful.

## Results

Six days of step data were analyzed for 142 subjects (male = 77, female = 65). Other quantitative demographic data can be found in Table 1.

**Table 1:** Overall demographics for all subjects (*n* = 142).

	**Age**	**Months Post-stroke**	**Mean daily steps**	**Self-selected speed (m/s)**
Average	63.25	51.53	4656.57	0.71
Standard Deviation	11.32	64.08	2952.86	0.21

In regards to the explored relationship between step counts and non-linear structure and complexity of activity, mean daily steps were found to be strongly related to JSD values (Figure 2A) with a linear regression (*p* < 0.001, *r*^2^ = 0.77). Further, mean daily steps were strongly related to participants' mean LZC values (Figure 2B). Total daily steps were also found to have a strong positive relationship with daily LZC values (Figure 2C). Quadratic regressions were used as more steps are correlated with more complexity, especially in the less active of stroke participants (*r*^2^ = 0.70 and *r*^2^ = 0.64, respectively).

**Figure 3:**
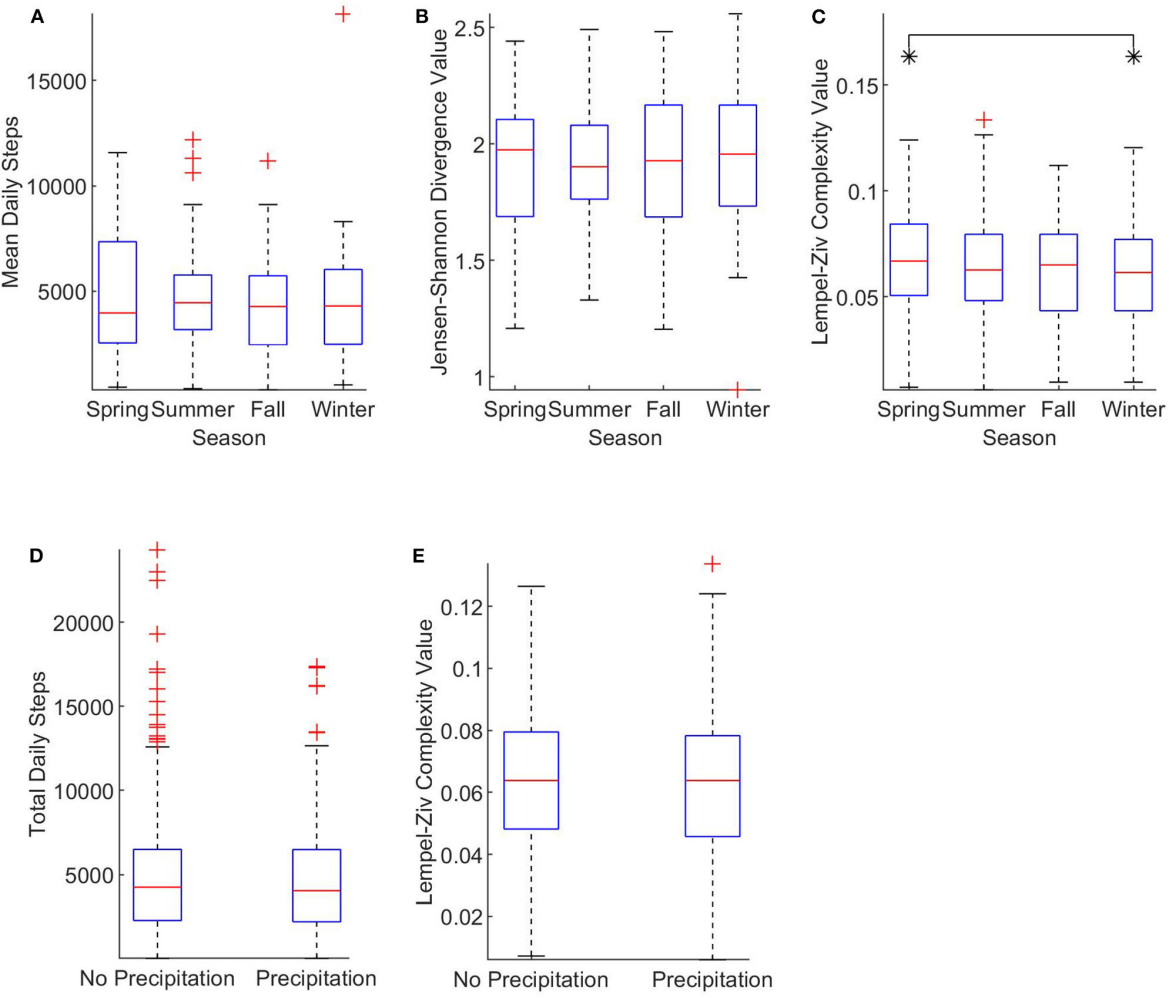
The effect of weather on linear and nonlinear physical activity measures. **(A)** Shows the effect of season on mean daily steps (*n* = 142). **(B)** Compares JSD values by season (*n* = 142). **(C)** Shows the effect of season on daily LZC values (*n* = 852). **(D)** Shows the effect of precipitation on total daily steps (*n* = 852). **(E)** Categorizes daily LZC values by precipitation presence (*n* = 852). All data is plotted on standard box and whisker plots with outliers notated with crosses (+). The edges of brackets annotated with an asterisk (*) indicate significant between-group differences, *p* < 0.05.

One-way ANOVA tests determined that mean daily steps (Figure 3A) and JSD values (Figure 3B) were not significantly affected by season (*p* = 0.92 and *p* = 0.98, respectively). However, a one-way ANOVA did find a significant effect on LZC values by season (Figure 3C). A follow-up Tukey test found that the significant effect was present with spring LZC values greater than winter LZC values (*p* = 0.019). Self-selected walking speed demographics by season can be found in Table 2. Additionally, one-way ANOVA tests found no significant effect of rain compared to no rain on total daily steps (Figure 3D) or LZC values (Figure 3E) (*p* = 0.35 and *p* = 0.81, respectively). With no effect of precipitation and a seasonal effect in just a single variable, we partially failed to reject our hypothesis that (1) activity amount, complexity, and structure is greater in favorable conditions and (2) activity amount, complexity, and structure is lesser in unfavorable conditions.

**Table 2:** Self-selected speeds based on seasons of individuals' data collection.

	**Self-selected speed (m/s)**
Spring Average	0.78
Spring Standard Deviation	0.19
Summer Average	0.70
Summer Standard Deviation	0.21
Fall Average	0.72
Fall Standard Deviation	0.20
Winter Average	0.66
Winter Standard Deviation	0.22

## Discussion

The goal of this investigation was to examine physical activity patterns and related factors in stroke survivors with the goal of informing further development of physical activity promotion programs that result in the greatest increase in physical activity levels. In our exploration of the relation of steps to structure and complexity, we confirmed a strong significant positive relationship, indicating that the non-linear measures JSD and LZC may be predictors of activity in stroke survivors. In analyzing these variables in relation to weather parameters, we found that we could partially fail to reject our hypothesis: in terms of complexity and season, we found that individuals' activity was more complex during spring than winter, but step counts and structure were not affected by season, and no variables were affected by precipitation.

Our investigation found that how active a stroke survivor is correlates to the similarity in their physical activity patterns and the complexity of their physical activity. It should be noted that it is mathematically possible for a person with a low mean daily steps value to also have a low JSD value (highly regular day-to-day patterns). However, our results show strong support that this does not occur in actuality as less active stroke survivors only behave with low regularity (high JSD values). Therefore, increasing the day-to-day structure of the least active of stroke survivors even slightly could increase their activity and potentially their health levels. It is possible that the present correlation has bidirectional causation. However, some patients may have greater results with instructions to build a regular, active schedule as opposed to a generic task to be more active. This information supports the idea that encouraging specific activities and routines to increase activity structure could help improve overall activity. Additionally, complexity of activity increased with total activity as LZC values increased as a function of step counts, so both ideas of structure and complexity should be combined in promoting daily physical activity. Designing habits of activities to do at certain times, while also including activity that requires a greater variance in walking intensity, may be able to increase structure, complexity, and therefore, the overall amount of activity that the stroke survivor is performing.

Our analysis found little effect of weather (season and precipitation) on physical activity after stroke. The only significant effect was an increase in activity complexity from winter to spring. Perhaps this means that stroke survivors are venturing outdoors more in the spring so that relaxed indoor sequences of activity are interspersed with sequences of activity at more intense rates, as required for community ambulation (Andrews et al., 2010). However, no relationship between season and steps indicates that more research is needed to determine if the significant step-complexity relationship could be utilized to motivate patients based on activity intensity as a way to increase activity amount during seasons in which weather may pose a barrier to activity. Interestingly, stroke survivors have been found to self-report “bad weather” as a barrier to the amount of physical activity performed, but especially the kind of unfavorable conditions that involve precipitation and increased risk of falling (Törnbom et al., 2017), but that relationship was not shown in our data; we did not see an effect of precipitation on walking activity. Perhaps the lack of precipitation relationship may mean the effect of weather may be more related to temperature; spring temperatures are mild, whereas winter temperatures are cold in the study population's region (Climate-Data.org). To answer some of the questions about the disconnect between our quantitative data and others' qualitative self-reported data, it would be interesting to perform a longitudinal study, to determine how the same individual is physically active across seasons and weather conditions. More specifically, a study that brings the quantitative aspects of the present study and the qualitative measures of previous studies together to compare how activity structure and complexity compare to any motivational reports of weather as an activity barrier would be especially valuable and warranted.

This is the first study to compare linear measures of physical activity after stroke to non-linear measures, like Jensen-Shannon Divergence, that can provide some insight into the pattern and complexity of physical activity after stroke. In stroke survivors specifically, this is the first study to use Lempel-Ziv Complexity in relation to physical activity after stroke. However, recent work has begun to use complexity measures to assess physical activity in other populations. For example, in “well-functioning older adults,” people with psychological concerns about falling had significantly lower complexity in their physical activity structure (Paraschiv-Ionescu et al., 2018). This knowledge is relevant to our investigation as stroke survivors report fear about falling (Törnbom et al., 2017), and this may contribute to an unhealthy decline in their physical activity complexity levels. Another study in healthy older adults, which was meant to validate an instrumented shoe, found that LZC was strongly related to the number of bouts of sustained walking (Moufawad El Achkar et al., 2016). This result suggests that one way to increase the number of sustained bouts of walking after stroke may be through increasing the complexity of daily walking structure. For example, electronic applications could be used to remind users to move, or change the intensity of their movement patterns, at varied times throughout the day as to avoid rigidity and increase complexity. These applications of LZC have developed from a variety of studies that investigate patterns, from those in DNA sequences to those in the activity of the brain in populations such as Alzheimer's disease and epilepsy (Aboy et al., 2006). The expansion of this measure across multiple disciplines is important to assess the varied patterns that innately occur both externally and internally in humans, which we know are important to health (Stergiou and Decker, 2011).

While previous research of the relationship between weather variables and physical activity is inconclusive, one study did find that physical activity amounts in community-dwelling older adults increased from winter to spring (Jones et al., 2017), similar to our result that complexity increased from winter to spring. However, our results disagree from that study in that we found no relationship between season and activity amount. One systematic review of the effect of weather on activity, while not completely conclusive, skewed toward weather having a significant effect and recommended alternative indoor activities during cold and wet months to compensate for the weather barrier, but no studies with stroke individuals were included (Tucker and Gilliland, 2007). In regards to previous studies relating weather to stroke survivors' activity, the perceived barrier of poor weather conditions, especially precipitation, to activity, as self-reported by individuals with stroke (Törnbom et al., 2017) largely disagrees with the present results, as no effect of precipitation was found, and only complexity, not amount or structure, of activity was found to be affected by season. Further research must be done to quantify a relationship between weather and the activity of those with stroke.

One weakness of this study is the 60 s sampling epoch (Knarr et al., 2013). The ability to analyze physical activity in smaller time epochs would allow a more precise picture of physical activity complexity and structure. Since our LZC measures were determined specifically from cadence levels, it is conceivable that more complex results, or more variances in the occurrences of physical activity patterns may be seen at a lower sampling epoch. It would be valuable to study how different sampling epochs affect complexity values to determine what epoch should be used in the future for the most valid results. It would also be interesting to observe how a narrower sampling frequency may cause a change in the relationship between JSD values, or the patterns of physical activity within the day across days, and physical activity amounts (Hurling et al., 2007; Fry and Neff, 2009).

An additional weakness of the present study is that information about whether or not participants were going outside or not each day was not collected, nor was their general attitude for, or against, being active during any given kind of weather. Thus, we cannot understand the full potential for weather to affect an individual's activity on a given day. Further research should investigate the effect of weather on the activity of stroke survivors including data about time spent outdoor compared to indoor. This data could be collected qualitatively through a questionnaire, asking participants if they believe certain weather conditions may be a barrier to their activity, where other conditions they feel more apt to be active. Additionally, brief daily summaries could be provided for basic descriptions of activity and overall weather conditions.

The present investigation found that non-linear measures Jensen-Shannon Divergence and Lempel-Ziv Complexity are valuable tools in analyzing the makeup of a stroke survivor's physical activity. Using JSD, we can directly relate a stroke person's total amount of physical activity to how regular their physical activity patterns are over multiple days. Using LZC, we found that stroke survivors that were more active overall, were performing walking activity of a greater variety, as measured by patterns of cadences. This study found that there may be a seasonal significance on physical activity, in terms of the complexity of activity, which are higher in the mild spring temperatures than in the cold winter, with precipitation not proving to have any effect. Longitudinal studies should be done to see if individuals adjust their own activity levels, timing, and type relative to precipitation and season and if such results are similar to those of this cross-sectional study. Further investigations into the effect of intervention types on physical activity levels and structure should also be done to determine if there are any existing causal relationships between linear and non-linear activity measures.

## Data Availability Statement

The data that support the findings of this study are available on request from the corresponding author, Brian A. Knarr. The data are not publicly available due to their containing information that could compromise the privacy of research participants.

## Ethics Statement

The study involving human participants were reviewed and approved by University of Delaware Human Subjects Review Board. The patients/participants provided their written informed consent to participate in this study.

## Author Contributions

DR and TW designed the study. TW participated in data acquisition. SA and BK analyzed the present data. All authors participated in data interpretation and were responsible for writing and approving the manuscript for submission.

## Conflict of Interest

The authors declare that the research was conducted in the absence of any commercial or financial relationships that could be construed as a potential conflict of interest.
